# Manipulating the Placebo Response in Experimental Pain by Altering Doctor’s Performance Style

**DOI:** 10.3389/fpsyg.2016.00874

**Published:** 2016-06-30

**Authors:** Efrat Czerniak, Anat Biegon, Amitai Ziv, Orit Karnieli-Miller, Mark Weiser, Uri Alon, Atay Citron

**Affiliations:** ^1^Sackler Faculty of Medicine, Tel Aviv UniversityTel Aviv, Israel; ^2^The Joseph Sagol Neuroscience Center, Sheba Medical CenterTel Hashomer, Israel; ^3^Department of Neurology, State University of New York at Stony Brook, Stony BrookNY, USA; ^4^Israel Center for Medical Simulation (MSR), Sheba Medical CenterTel Hashomer, Israel; ^5^Department of Psychiatry, Sheba Medical CenterTel Hashomer, Israel; ^6^Department of Molecular and Cell Biology, Weizmann Institute of ScienceRehovot, Israel; ^7^The Theatre Laboratory, Weizmann Institute of ScienceRehovot, Israel; ^8^Theatre Department, University of HaifaHaifa, Israel

**Keywords:** placebo, placebo response, placebo responder, placebo analgesia, performance style, doctor–patient interaction, reward expectation, pain

## Abstract

**Background:** Performance is paramount in traditional healing rituals. From a Western perspective, such performative behavior can be understood principally as inducing patients’ faith in the performer’s supernatural healing powers and effecting positive changes through the same mechanisms attributed to the placebo response, which is defined as improvement of clinical outcome in individuals receiving inactive treatment. Here we examined the possibility of using theatrical performance tools, including stage directions and scripting, to reproducibly manipulate the style and content of a simulated doctor–patient encounter and influence the placebo response in experimental pain.

**Methods:** A total of 122 healthy volunteers (18–45 years, 76 men) exposed to experimental pain (the cold pressor test) were assessed for pain threshold and tolerance before and after receiving a placebo cream from a “doctor” impersonated by a trained actor. The actor alternated between two distinct scripts and stage directions, i.e., performance styles created by a theater director/playwright, one emulating a standard doctor–patient encounter (scenario A) and the other emphasizing attentiveness and strong suggestion, elements also present in ritual healing (scenario B). The placebo response size was calculated as the %difference in pain threshold and tolerance after exposure relative to baseline. In addition, subjects demonstrating a ≥30% increase in pain threshold or tolerance relative to baseline were defined as responders. Each encounter was videotaped in its entirety.

**Results:** Inspection of the videotapes confirmed the reproducibility and consistency of the distinct scenarios enacted by the “doctor”-performer. Furthermore, scenario B resulted in a significant increase in pain threshold relative to scenario A. Interestingly, this increase derived from the placebo responder subgroup; as shown by two-way analysis of variance (performance style, *F* = 4.30; *p* = 0.040; η^2^ = 0.035; style × responder status interaction term, *F* = 5.21; *p* = 0.024) followed by post hoc analysis showing a ∼60% increase in pain threshold in responders exposed to scenario B (*p* = 0.020).

**Conclusion:** These results support the hypothesis that structured manipulation of physician’s verbal and non-verbal performance, designed to build rapport and increase faith in treatment, is feasible and may have a significant beneficial effect on the size of the response to placebo analgesia. They also demonstrate that subjects, who are not susceptible to placebo, are also not susceptible to performance style.

## Introduction

Performance is paramount in traditional healing rituals. Besides being expert healers, shamans, and medicine men are trained performers (Kendall, 1996). Their performances consist of elements such as drumming, chanting, singing, dancing, role playing while in trance, ventriloquism, magic, acrobatics, comedy, and more (Kirby, 1975; Lee, 1990). These acts are usually performed publicly in symbolic costumes and often with masks, fans, rattles, and other props (Eliade, 1964; Vitebsky, 1995; Moffitt Cook, 1997). Although traditional healers may use herbs and other pharmacologically active ingredients, they never administer them without the appropriate performance (Vitebsky, 1995). From a Western perspective, such performative behavior can be understood principally as inducing patients’ faith in the performer’s supernatural healing powers (Winkelman, 2000). Faith leads to an expectation of a successful outcome, which supposedly triggers a self-healing mechanism in the patient (Achterberg, 1985).

Western medicine, relying on scientific and technological developments in surgery and pharmacology, has moved away from healing through performance. Still, patients’ faith in their physician’s ability to heal may have an important role in the process of recovery and should probably not be underestimated. Randomized placebo-controlled trials have demonstrated that a substantial percentage (20–70%) of patients respond to placebo treatment (Montgomery, 1999; Rutherford et al., 2014), and that this positive response is based on patients’ expectation to recover (de la Fuente-Fernandez and Stoessl, 2002; de la Fuente-Fernandez, 2009). Studies have also shown that this expectation can be enhanced by an effective doctor’s performance (Kaptchuk, 2002; Kaptchuk et al., 2008; Kelley et al., 2009). Results of placebo research and studies on the quality of doctor–patient encounters may thus lead to a re-evaluation of performance elements in the practice of evidence-based medicine and to the introduction of systematic performance training into medical education in the future. Naturally, the performance style suitable to the modern clinic and hospital environment is distinctly different from that of the traditional healers mentioned above, but the goals and the principles of the two distinct styles could be similar.

The history of placebo is intertwined with the history of medicine (Shapiro and Shapiro, 1997; also reviewed in Czerniak and Davidson, 2012). The “placebo response” is defined as a positive outcome in patients who have received placebo (a non-specific intervention), beyond changes due to the natural course of disease. The substantial and increasing placebo response poses a problem for the pharmaceutical industry, as it increases the difficulty of identifying drug–placebo differences for new drugs in development (Lakoff, 2002; Kemp et al., 2010) especially in the neuropsychiatric field (Walsh et al., 2002; Stein et al., 2006; Cappelleri et al., 2009; Muthen and Brown, 2009; Wasan et al., 2010). Conversely, the placebo response may be viewed as beneficial from the patient perspective, possibly allowing dose reduction or even replacement of active drugs with placebo in susceptible individuals and reducing the rate of adverse effects (Hilsden and Verhoef, 1999; Tamura and Huang, 2007).

While it has been suggested that placebo responses may be due to spontaneous remission of the disease, statistical regression to the mean, effects of co-intervention or patients’ will to please their doctor (Kienle and Kiene, 1997), recent findings suggest a major contribution of brain circuits and neurotransmitters involved in the expectation of reward (Benedetti, 2009). The degree of placebo responsiveness may in part result from the different effect that anticipation has on the reward system in each individual. The brain reward system consists of neuronal circuits involving cognitive, emotional, and motor processes, which are often studied in relation to natural rewards such as food or to monetary prizes (Mogenson and Yang, 1991; Kalivas et al., 1999; Scott et al., 2007). The nucleus accumbens, a nucleus of the basal ganglia occupying the ventral extent of the striatum, has long been recognized as a neural core of the reward system (Berridge and Robinson, 1998; Cardinal et al., 2002) and linked with motivated behavior (Salamone et al., 2005). In a positron emission tomography (PET) study of subjects with Parkinson’s disease, de la Fuente-Fernandez and colleagues (de la Fuente-Fernandez et al., 2001; de la Fuente-Fernandez and Stoessl, 2002) showed release of endogenous dopamine in the striatum following the administration of placebo. No relationship was found between dopamine activity in the nucleus accumbens and placebo effect on motor function, suggesting dopamine release was indeed related to the expectation of reward.

On this background, manipulations of the patient–doctor interaction designed to increase expectation of reward are likely to have a beneficial effect on treatment response, including response to placebo. Historically, research on the factors which enhance placebo response has focused on attributes of the treatment, showing that more invasive treatment modalities (Meissner et al., 2013) or specific pill colors (Moerman, 2002) are associated with higher placebo response rate. The act of suggestion within medical treatment, often in the form of verbal suggestion, was more recently investigated in several studies which supported its contribution to the size of the placebo response. For instance, a study in healthy adults who were restricted to a night of 5 h sleep and then given decaffeinated coffee, showed that the group which was under the impression it received caffeinated coffee performed better (Anderson and Horne, 2008). More recent studies on skin reactions in healthy volunteers, demonstrated that verbal suggestion had a positive impact on the experience of itch (Darragh et al., 2015; Bartels et al., 2016), most likely engaging the same brain networks (Hashmi et al., 2014). There have been fewer studies on the impact of other verbal and non-verbal performative elements of the context in which the placebo is administered, including the interpersonal communication style, doctor’s appearance and actions. In these studies both interaction style and verbal suggestion were shown to contribute to the placebo response size in patients with irritable bowel syndrome (Kaptchuk et al., 2008; Kelley et al., 2009; Hall et al., 2012).

The behavior in the doctor–patient encounter can be perceived as “performance in everyday life”. Goffman’s seminal work, *The Presentation of Self in Everyday Life* (Goffman, 1959) led sociologists, anthropologists, and performance studies scholars to study performance in everyday life (in the work place, in the political arena, in education, public health system, etc.) in terms that are quite similar to those used in the analysis of theatrical performance (Turner, 1982; Schechner, 1985). More recently, the founders of the field of Social Performance Studies have offered acting training to professionals in the electronic media and tourism businesses in China in order to improve their interpersonal skills and achieve better customer satisfaction rating (Sun and Fei, 2014). A theater improvisation seminar was offered to students at Northwestern University’s Feinberg School of Medicine under the title “medical improv” with the purpose of preparing and training them to the unpredictable aspects of a doctor–patient encounter (Watson, 2011). We are not aware, however, of published studies using theatrical performance tools in the context of the placebo response. Several important issues are not addressed in the available literature, e.g., the effects of structured manipulation of verbal and non-verbal aspects of the doctor–patient interaction on the placebo response size as well as the placebo response rate, defined as the proportion of subjects (placebo responders) exhibiting a clinically meaningful improvement.

The possibility that placebo responders share specific genetic and personality traits has been addressed by several recent studies in healthy volunteers and subjects suffering from various disorders (Leuchter et al., 2009; Hall et al., 2012; Pecina et al., 2015). In this context, it has been suggested that the doctor–patient interaction style has a more robust effect on the size of the placebo response in subjects with the genetic profile associated with placebo responders (Hall et al., 2012).

One population which stands to gain the most significant benefit from full or partial placebo-for-drug treatment replacement are healthy subjects with acute pain (e.g., following dental procedures). This situation can be emulated by using experimental pain paradigms such as the cold pressor test (CPT; Hines and Brown, 1936; Lovallo, 1975). Subjects afflicted with chronic pain (Pergolizzi et al., 2008; Dzau and Pizzo, 2014) or other disorders commonly treated with potentially addictive or toxic drugs can also benefit from dose reduction or placebo replacement. As a first step toward addressing these questions, we applied our knowledge from the field of communication in medicine and used theatrical performance tools to examine the hypothesis that structured manipulation of the doctor–patient interaction can be applied consistently and have measurable effects on the placebo response in experimental pain, with more robust effects expected in placebo responders.

## Materials and Methods

### Study Population

One hundred and fifty-five healthy volunteers (men and women 18–45 years old), were recruited from local universities, colleges, and social networks through fliers and social media. Potential subjects were told they would be participating in an evaluation of new analgesics, when they were actually administered placebo by an actor impersonating a physician. Subjects who were interested in participating were scheduled and emailed the informed consent at least 24 h prior to their assessment. Upon arrival, the subjects received information on the different stages of the study and gave their written consent to participate, as well as to be videotaped during the experiment. The subjects were blind as to the objectives of the study, which were spelled out in the protocol but not in the informed consent. This was done with the approval of the local institutional review board (IRB) as well as by Israel’s Ministry of Health ethics committee, after both were convinced that full disclosure in this case would have no bearing on the (minimal) risk involved (i.e., temporary pain) but will jeopardize the achievement of the study’s aims. Healthy volunteers were excluded if they had diabetes, chronic pain, cardiovascular disease, fainting spells, frostbite, Raynaud’s syndrome, or a cut/open wound/fracture in the dominant hand.

### Development of Two Scenarios

Two different scenarios were tested (scenario A and scenario B). Both included a verbal component (doctor’s part of the dialog with the volunteer) and a non-verbal component (doctor’s mise-en-scène, i.e., his gesticulation, his movement in the room, its pace and its relation to the volunteer). The scenarios were scripted to be of the same length (4.5–6.5 min) and similar basic structure: opening, short interview, hand examination, explanation about the pain-relief cream and how to apply it, and closing. Scenarios A and B took place alternately and randomly at the same doctor’s clinic at the Israel Center for Medical Simulation (MSR), Sheba Medical Center.

The performance text (verbal and non-verbal content) of scenario A was derived from personal recollections of the investigators as patients in the Israeli public health system and from video documentation of doctor–patient encounters from the MSR archives. The verbal part was laconic and functional. The stage directions indicated restricted movement of the actor/doctor, who remained seated at his desk throughout the encounter, continuous reading of text on the computer screen, typing, minimal eye contact, and lack of tactile interaction with the volunteer. The purpose was to simulate the experience of a routine visit to a doctor’s office in a public health clinic.

The performance text of scenario B was based on healing rituals performed by shamans and healers as observed and documented by the corresponding author, who is a theater director (AC) during 20 years in different parts of the world, as well as on other available video documentation of such healing rituals. The material was adapted to the study population by omitting culture-bound elements that might seem exotic and out of place to a Western subject (e.g., the use of elaborate costuming, dancing, chanting, and references to spirits). The verbal part was personal, attentive to the volunteer, and used imagery in the questions and the explanations. The stage directions indicated free movement in the room, including periods of standing while speaking, frequent eye contact, and deliberate tactile interaction with the volunteer (handshake while greeting and touching of the hand while examining it after the experimental pain procedure). The purpose was to establish the subjects’ faith in the doctor and in the remedy he prescribes by means of his performance style.

### Casting

The same actor portrayed the physician in both scenarios in order to control for variables such as physical appearance or timber of voice. For credibility purposes, the actor portraying the physician was typecast to match a common doctor’s stereotype: middle-aged Caucasian man, slim, height slightly above average, wearing glasses, short-haired, and clean-shaven. The particular actor was chosen also because he is not a popular mainstream actor who could be recognized by the participating volunteers.

### Costume and Props

In both scenarios, the actor wore an unbuttoned white coat over dress pants and shirt with no necktie, as is the custom in Israel. A photo ID was attached to the coat’s left breast pocket. A cellular phone was placed in his pants’ pocket and was used once in scenario A. A laptop computer was placed in front of him on the desk. Two large posters illustrating pain pathways and analgesic mechanism were hanged on the wall behind the desk. In the top drawer of a chest behind the doctor’s chair and to his right, were placed several standard pharmaceutical plastic containers containing odorless white moisturizer (the “cream”). The jars were labeled “#AB221” or “#EC499.”

### Rehearsals and Pilots

Rehearsals with the actor portraying the physician led to the second phase of the development of the two scenarios. With two investigators alternately playing volunteers of various characters, the actor gained confidence and developed a credible presence of an experienced physician. His input led to changes in the verbal components of the scenarios, making his part of the dialog more fluent and persuasive. The scenarios’ final version emerged from a pilot in which volunteers (students and lab staff) who had not been exposed to experimental pain, participated in the dyadic encounters with the actor playing the doctor and offered their feedback to the research team. Consequently, the scenarios were modified (example: in the original scenario B, the doctor applied the cream to the volunteer’s hand, but in the final version it was the volunteer him/herself who did it because several female participants in the pilot felt uncomfortable with the doctor’s touch).

### Setting of the Study

All assessments took place at MSR, Israel’s national resource for Simulation-based Medical Education, located at the Sheba Medical Center. Designed as a virtual health care environment, this 2000 m^2^ center focuses on patient safety and quality care training of health professionals. MSR utilizes simulation modalities, including high-tech procedural simulators as well as role-playing actors to improve both clinical and communication skills. The center is equipped with one-way observation booths and an audio-visual recording system for the purpose of formative training and video-based debriefing as well as for performance testing (Ziv et al., 2006).

This facility allowed us to test up to five participants at the same time, occupying four rooms simultaneously: the pain experiment took place in room 1 which was set up as a standard examination room with a digital timer not visible to the subject. Room 2 contained computers loaded with the personality questionnaires for the subjects to complete. Room 3 was the “doctor’s office,” furnished to look like a standard doctor’s office. This room was also equipped with two video cameras, one offering a bird’s eye view perspective of the room and the other situated to show the actor and the subject from the side. Of several angles used for the side camera, the one most useful for automated video analysis reported in a parallel publication (Hart et al., submitted) was at 1 m horizontal distance and a 1.7 m height. Subjects then moved on to room 4 which was set up as a standard examination room and contained mouthwash and test tubes for DNA collection. This room also hosted the cognitive assessment which was completed with the help of an assistant.

### Experimental Design

**FIGURE 1:**
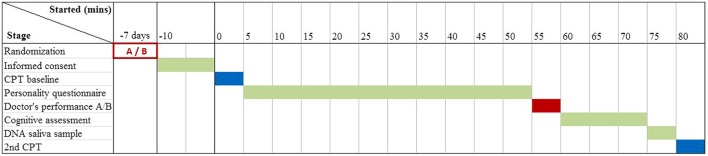
**Experimental design.** CPT, cold pressor test; A/B, performance scenarios.

Subjects were scheduled in groups of 10–16/day. The subjects in each group were randomized to one of two scenarios (code named A and B by the actor), using Java “Collections.shuffle” command. The code was opened only at the end of the data collection phase, so that the investigators scheduling subjects and administering the other tests were blind to the nature of the scenario the subjects were exposed to. In addition, the subjects were blind to the fact that they were randomized to two scenarios. The subjects underwent a pain test (CPT) and filled a personality questionnaire. Then they experienced a ∼5-min encounter with a professional actor impersonating a pain specialist, who gave them an over-the-counter, odorless hand moisturizer, which they believed to be an analgesic. Next they completed some cognitive tests and gave a saliva sample for DNA analysis. Finally the subjects repeated the CPT (**Figure 1**).

### Cold Pressor Test

The CPT (Hines and Brown, 1936; Lovallo, 1975) is a well-established acute pain paradigm in which subjects are asked to immerse their dominant hand in cold water (a 4 ± 0.5°C iced-water bath) and hold it in steadily with the fingers spread out. A hidden stopwatch is activated from immersion and until the hand is withdrawn. The subject is asked to state the exact moment at which the cold stimulus starts to elicit pain (i.e., pain threshold, measured in seconds) and then is instructed to withdraw his/her hand when the pain becomes intolerable. The time from immersion to withdrawal is recorded as the pain tolerance (in seconds). A maximal 5-min period (300 s) is set for safety reasons, and subjects who did not withdraw their hand up to this point are instructed to do so.

### Placebo Response Assessment

The placebo response size was defined as the percent of change between the second CPT (2nd CPT) threshold or tolerance parameters and those at baseline (CPT bl), i.e., % Response$=\frac{2nd\,\;CPT-CPT\,\;bl}{CPT\,\;bl}\times 100$. A positive placebo response was defined as a response of at least 30% over baseline, and a placebo responder is a subject demonstrating it.

### Statistical Analysis

Based on power analysis of previously published studies (Waber et al., 2008; Doehring et al., 2011; Treister et al., 2013), we estimated that a total sample size of 120 subjects was needed in order to detect a medium size effect (0.5–0.6 Cohen’s *d*, Cohen, 1988) of doctor’s performance on the placebo response size, with 0.8 power. We used Student’s *t*-test to study whether there were significant demographic (age and education level) differences between the groups exposed to the two performance styles. Pearson’s correlations were implemented in order to test possible correlations of age and education level with the mean response size. One-way analysis of variance (ANOVA) was used to test the response rates and mean response size in CPT threshold and tolerance measures, and the distribution of response rates across gender and performance styles were tested using Pearson’s χ^2^. We applied a two-way ANOVA in order to test potential effects of performance styles and responsiveness (being a placebo responder vs. a non-responder) on the mean response size. All statistical analyses were carried out using SPSS Software, version 22.

## Results

### Scenario Development

#### Scenario A

The doctor busies himself typing on his laptop just before the volunteer knocks on the door. He says “come in” without looking at the person entering and without greeting that person unless the volunteer greets first, in which case the doctor responds with a greeting. He motions to the volunteer to sit down across the desk as he continues to type, his eyes still on the computer screen. Typing, which continues for another minute, is interrupted by the ringing of the doctor’s cell phone. He takes it out of his pocket, studies the screen, and shuts it off, replacing it in his pocket. He continues to type for a few more seconds, then asks the volunteer for his/her name and finds it in his computer files. Now he looks at the volunteer for the first time and asks if he/she has just gone through the CPT. He asks to have a look at the hand that was immersed in the ice water, examines it visually from across the desk, asks to see the back of the hand and examines it briefly as well. He asks the volunteer what made him volunteer for the study and pretends to type the answer (payment/a friend’s recommendation, etc.) on his laptop. He then rolls on his chair toward a chest of drawers, opens the top drawer and pulls out a small plastic jar containing moisturizer cream, stating that this new pain relief medicine is being tested and the volunteer should apply it evenly on both sides of the hand that has been in the ice water. He oversees the action performed and then asks the volunteer to proceed to the other room where the 2nd CPT round will take place.

#### Scenario B

When there is a knock on the door, the doctor rises, walks toward the door, greets the volunteer by name, shakes his/her hand (except for orthodox women) and invites him/her to come in. He asks the volunteer to sit down across the desk and takes his seat on the other side. He asks what made the volunteer participate in the study and types the answer quickly on his laptop, resuming eye contact with the volunteer right away. He asks about the volunteer’s experience during the CPT, listens to the answer and repeats it. He asks to examine the hand that was in the ice water, takes it in his own hand, and looks at it carefully while touching it on both sides. He then asks the volunteer to describe the pain he has just experienced, guiding him/her to use a metaphor or an image to communicate the particular feeling (e.g., like a knife cutting the flesh or like a burn). He continues by asking how the volunteer normally deals with pain. He listens to the answer and reflects it briefly, then proceeds to say that as a doctor, he has been studying pain and people’s reactions to it for many years and has come to the conclusion that pain is a very personal experience that calls for a treatment that is designed individually for each person suffering from it. He says that this is the approach used in the present study, and that the new pain relief cream being tested is the product of many years of research in both Western and complementary medicine. He adds that the cream has different formulae, each designed for a different type of personality. He looks at the computer screen, as if studying the volunteer’s answers to the questionnaire, and while rising from his seat, says that according to the answers he has just read, the type of cream that would work most efficiently in this specific case, would be… He pauses before completing the sentence, stands up, turns to open the top drawer and carefully chooses one of the many plastic jars in it. He does this with his back turned to the seated volunteer. He then turns around, holding the jar above his head and hands it to the volunteer with a large gesture. He explains that the cream should be evenly applied on both sides of the hand, and adds that he is convinced that it is going to be very effective in reducing the pain during the second CPT. He then escorts the volunteer to the door.

**FIGURE 2:**
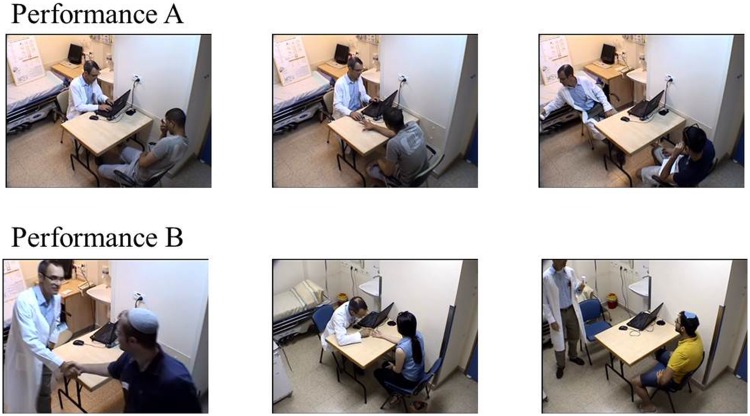
**Representative illustrations from scenarios A and B.** Top row – scenario A, bottom row – scenario B. Left panels: “doctor” greeting subject. Middle panels: Inspection of the hand. Right panels: Presentation of the medication.

The final scenarios utilized in the study are illustrated in **Figure 2.** The full scripts and stage directions can be found in the Supplementary Material.

### Cold Pressor Test

In the first 3 days of testing, the exposure time was limited to 180 s and there were technical problems with maintaining the temperature in the desired range. The protocol was then amended to include a 300 s exposure and consequently we did not use the data from the subjects (*N* = 33) tested in the first 3 days and continued enrollment until we had 122 subjects. The groups assigned to the two performance styles were reasonably matched for age, sex, and education level (**Table 1**).

**Table 1:** Subject demographics by scenario.

	Scenario A	Scenario B
*N*	60	62
Women (%)	16 (27)	30 (48)
Age (year, mean ± SE)	26.5 ± 7^∗^	23.9 ± 6
Education (year, mean ± SE)	14.0 ± 2^∗^	13.4 ± 2


*^∗^*p* < 0.05, Student’s *t*-test, two-tailed.*

### Demographic and Baseline Characteristics of CPT Measures

Student’s *t*-test showed small but statistically significant difference in age (years, A: 26.5 ± 7; B: 23.9 ± 6; *p* = 0.025) and in education level (A: 14.0 ± 2, B: 13.4 ± 2, *p* = 0.044) between scenario “A” and “B” groups (**Table 1**). However, Pearson’s correlation analysis showed that CPT threshold and tolerance response sizes were not significantly correlated with either age (*r* = 0.11, *p* = 0.25 and *r* = -0.01, *p* = 0.88, respectively) or education level (*r* = -0.12, *p* = 0.21 and *r* = -0.11, *p* = 0.24, respectively).

The baseline mean values (in seconds) of CPT threshold and tolerance in men and women are shown in **Table 2.** We found significantly higher mean threshold for men (20.7 ± 2) relative to women (12.1 ± 1) (*t* = 3.231, *p* = 0.002), as well as significantly higher mean tolerance for men (92.9 ± 12) relative to women (49.5 ± 8, *p* = 0.005). These differences, which were expected based on the literature (Kowalczyk et al., 2006; Sinha and Dubey, 2015) supported our a priori decision to express changes due to placebo and performance style as %change rather than an absolute difference in seconds.

**Table 2:** Baseline pain threshold and tolerance measures across gender.

	CPT threshold (seconds)			CPT tolerance (seconds)		
		
	*N*	Baseline	%Change	*N*	Baseline	%Change
Men	76	20.7 ± 2^∗∗^	-1.3 ± 9	49	92.9 ± 12^∗∗^	-0.5 ± 9
Women	46	12.1 ± 1	-25.3 ± 6	41	49.5 ± 8	-20.0 ± 5


*Results are presented in mean ± standard error of *N* subjects. ^∗∗^*p* < 0.01, Student’s *t*-test, two-tailed, relative to women.*

### Placebo Response

Based on CPT threshold at baseline and after placebo administration, we calculated the response rates and the mean response size in men and women. The response rate was 16% in men and 13% in women (*p* = 0.80), however, men showed a trend toward a larger response size relative to women (*F* = 3.60, *p* = 0.060).

When evaluating response rates and the mean response size for CPT tolerance, we needed to exclude all subjects who held their hand in the ice water for the maximum time (300 s) at baseline and after treatment due to a ceiling effect (*N* = 33). Using this parameter and population, response rate was 18% in men and only 7% in women (*p* = 0.21). Men again showed a trend toward a larger response size relative to women (*F* = 3.57, *p* = 0.062; **Table 2**).

The number of responders defined as those showing a 30% increase in pain threshold was 7 in scenario A and 11 in scenario B. The difference was not statistically significant. In addition, subjects in scenario B showed a higher mean response size than subjects in scenario A. The number of responders in the tolerance parameter was larger in scenario B (scenario A, *N* = 3; scenario B, *N* = 9) but the difference was not statistically significant (χ^2^ = 2.88; *p* = 0.123). Subjects in scenario B again showed a higher mean response size than subjects in scenario A (**Table 3**).

**Table 3:** Performance style and placebo response.

	CPT threshold (seconds)			CPT tolerance (seconds)		
		
	*N*	Response size	Response rate	*N*	Response size	Response rate
Scenario A	60	-17.4 ± 6	7 (12%)	43	-14.5 ± 8	3 (7%)
Scenario B	62	-3.6 ± 11	11 (18%)	47	-4.7 ± 7	9 (19%)


*Response size is presented as mean ± standard error of *N* subjects. Response rates are presented as the absolute number and (%).*

**FIGURE 3:**
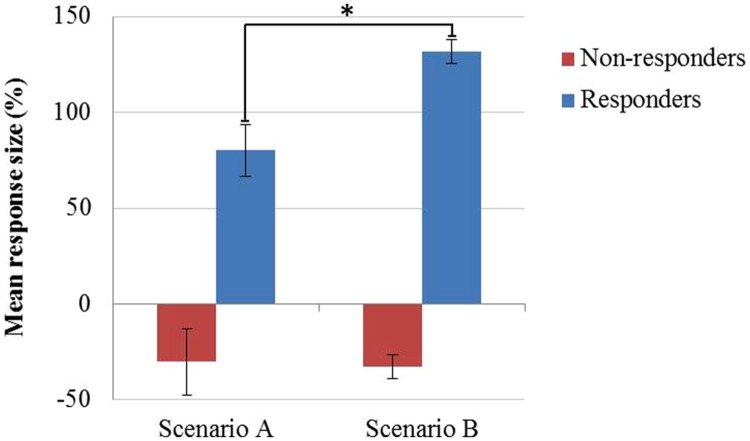
**Performance style and response size in placebo responders and non-responders.** Bars represent mean ± SE of % change in CPT threshold of 60 subjects in scenario A: 53 non-responders vs. 7 responders and 62 subjects in scenario B: 51 non-responders and 11 responders. Two-way ANOVA by performance style and responsiveness revealed significant effects of doctor’s performance (*F* = 4.30; *p* = 0.040; η^2^ = 0.035) and responsiveness (*F* = 134.71; *p* < 0.001) as well as a significant interaction term (*F* = 5.21; *p* = 0.024). ^∗^*p* = 0.020, Fisher’s least significant difference *post hoc* test.

Next we evaluated the impact of responsiveness and the two performance styles on the placebo response size for CPT threshold. A two-way ANOVA revealed a significant effect of responsiveness (*F* = 134.71, *p* < 0.001) and a significant effect of performance style (*F* = 4.30, *p* = 0.040; η^2^ = 0.035) as well as a significant interaction term (*F* = 5.21, *p* = 0.024). Fisher’s least significant difference *post hoc* analysis revealed that the effect of performance style originated in the responder group; with responders exposed to scenario B demonstrating larger response size (132 ± 38 s) than responders in “A” group (80 ± 22 s, *p* = 0.020). In contrast, there was no effect of performance style on response size in non-responders (**Figure 3**). A two-way ANOVA of the response size in CPT tolerance revealed a trend-level effect of performance style (*F* = 1.89, *p* = 0.173) and a trend-level interaction term (*F* = 1.73, *p* = 0.192).

## Discussion

Our study demonstrates that it is possible to construct reproducible performance scenarios for doctor–patient encounters using theatrical performance tools, which can significantly alter pain threshold in a subgroup of subjects exposed to experimental pain. Using the CPT, a common and validated experimental pain paradigm, we show that exposure to a performance style incorporating verbal and non-verbal elements of engagement as well as authority and suggestion adapted from traditional healing rituals (scenario B) results in a significant increase in the size of the positive response to administration of a placebo drug; relative to a performance style entailing a relatively impersonal and curt communication emulating a standard clinic doctor visit in the Israeli public health system.

Interestingly, this effect was limited to the subgroup of subjects who were placebo responders as defined by a fairly conservative criterion; i.e., subjects who responded to the placebo by an increase in pain threshold of 30% or more. This criterion was adopted from the pain literature since it reflects a response, which would be clinically meaningful in a real life or clinical trial setting (Cappelleri et al., 2009; Wasan et al., 2010). This approach is similar to the one adopted by Meissner et al. (2013), in which a 50% reduction in attack frequency was considered a placebo response in migraine sufferers. We also tested whether the developed scenarios could be objectively distinguished using an automated video analysis on the motion of doctor–patient dyad. We found significant differences in motion between the two scenarios, with more synchrony in scenario B than A (Hart et al., submitted).

Although, to our knowledge, this is the first study to employ theatrical performance tools to manipulate the size of the placebo response, the results do resonate with the published literature showing that this parameter, as well as patients’ response to active intervention in the clinical setting, can be manipulated by verbal suggestion and communication style (Hall et al., 2012; Darragh et al., 2015; Bartels et al., 2016). This study was carried out relying on the notion that a placebo responder is an individual who bears specific characteristics or a profile, which consist of personality traits, cognitive preconditioning and genetic predisposition to placebo responsiveness. Waber et al. (2008) exposed subjects to either “regular priced” or “low priced” placebo drug, studying the contribution of their vulnerability to commercial features or expectation to the placebo response. A different group tested the impact of three treatment methods: waiting-list, standard and augmented (with warmth and attention) interaction style on the placebo response in irritable bowel syndrome, and found that the compassionate and attentive style was associated with the best response to treatment (waiting-list < standard < augmented; Kaptchuk et al., 2008). The same group later found that placebo response size was dependent on the number of methionine alleles in Catechol-O-methyl transferase val158met (val/val < val/met < met/met) indicating the contribution of genotype to the placebo response (Hall et al., 2012).

The percentage of the study population who showed a placebo response in our study (15% overall) is at the low end of the range (20–70%) reported in the recent literature (Lakoff, 2002; Rutherford et al., 2014). This may be due to the fact that the placebo intervention we used was extremely benign (hand lotion); as it is known that the effectiveness of placebo is directly correlated with its aversive properties (i.e., pill < injection < surgery, and bitter, large and/or multiple pills > a single sugar pill; e.g., Rogers et al., 2014; Ross et al., 2015). Another, possibly related limitation of our study is the smaller number of women in the group randomized to scenario A relative to scenario B; although the study was not powered to show a sex difference in the response to performance style. Although gender was shown not to be a significant variable in placebo studies in depression (Stein et al., 2006; Weimer et al., 2015), such differences may or may not occur in other clinical indications and experimental paradigms.

In this regard, we did observe the well-known difference in pain threshold between men and women, whereby women had significantly lower thresholds at baseline relative to men (Kowalczyk et al., 2006; Sinha and Dubey, 2015) and women in our study also showed a smaller response to placebo, with the majority actually showing a nocebo effect. We note that the effects on CPT tolerance in our study, though similar in direction to the effects on threshold, did not reach statistical significance. This is most probably due to statistical power issues, since 26% of the subjects showed ceiling effects at baseline (keeping their hand in the ice water bath for the maximum of 300 s) and thus did not contribute useful data to the analysis of a positive placebo response.

Additional studies using pills or injections as placebo, stratifying subjects by sex prior to randomization and using lower temperatures need to be carried out to answer these questions. Replication of our findings in clinical populations; employing professional physicians of both sexes, are necessary in order to establish their generality and possible application in medical training, with the aim of improving patient outcome across diseases and treatment modalities. Future studies using performance tools in clinical trial settings could demonstrate the potential of borrowing performance principles and techniques from traditional healing and applying them to physician–patient encounters in Western medicine, following certain necessary modifications. Performance tools could thus eventually be incorporated into the systematic training of physicians and medical students, possibly to complement programs in Narrative Medicine (Charon et al., 2016) and Relational Medicine (Raia and Deng, 2014).

## Author Contributions

EC, AB, UA, AC, AZ, and OK-M contributed to the conception and design of the work. EC was responsible for data acquisition. EC, AB, UA, and AC interpreted the data and EC and AB did the analyses. EC, AB, UA, AC, AZ, OK-M, and MW drafted and revised the manuscript which was then finalized by EC, AB, and AC.

## Conflict of Interest Statement

The authors declare that the research was conducted in the absence of any commercial or financial relationships that could be construed as a potential conflict of interest.
