# Supplementary material for: Manipulating the Placebo Response in Experimental Pain by Altering Doctor’s Performance Style
Source: Front Psychol. 2016 Jun 30;7:874. doi: 10.3389/fpsyg.2016.00874 (PMC4928147; doi:10.3389/fpsyg.2016.00874)
Supplement: Supplementary file 1 [file Data_Sheet_1.DOCX]

Supplementary Material

Manipulating the Placebo Response in Experimental Pain by Altering Doctor’s Performance Style

Efrat Czerniak, Anat Biegon, Amitai Ziv, Orit Karnieli-Miller, Mark Weiser, Uri Alon, Atay Citron*

*** Corresponding Author:** Atay Citron, PhD aticitron@gmail.com

# Scenario A

As the volunteer (V) enters the room, the “physician” (P) will be busy typing on the computer, when the screen is turned away from V sitting in front of him. If V greets P, P will respond without getting up or taking his eyes off the screen. P will not greet V in advance, but if V waits to be seated, P will make a gesture with his hand. Throughout most of the encounter P will be busy typing different details, filling the patient’s forms etc. Their eye-contact will be limited to the moments when P addresses V with direct questions or gives him/ her instructions.

P: Name?

V:

P: Did you go through the pain threshold and tolerance test? (typing on the computer)

V:

P: (continues typing, raises his eyes to V) Show me your hand. Is this the hand dipped in the ice water? (continues typing). Anything special or is everything O.K.?

V:

P: (typing) Are you ready to continue with the experiment? (typing)

V:

P: I will go over your details (types, stops and then refers to V) You will now receive a pain relieving cream. Apply it on the hand which was dipped in the ice water earlier. We are testing the effect of the cream.

(if asked by V, P gives a short explanation on the cream’s mechanism, while pointing out from his seat towards the poster. If no explanation is expected, P does not give one)

P: (holds the cream in his hand and feeds the label details into the computer. He then offers the cream to V) This is the cream. Apply it on your palm, between your fingers too, (glimpses at V’s application of the cream, making sure V follows instructions). Wait for five minutes and then you will be asked to enter the room where the next experiment takes place.

Thanks and good luck.

V exits the room.

**Scenario B**

As the volunteer (V) enters the room, the “physician” (P) rises from his chair and greets the volunteer, inviting him/her to sit on the vacant chair at the desk.

P: Hello, you are (name of volunteer, which he has learned from…). My name is Dr. J. (gives his full name and allows the volunteer to respond to the greeting), and I will give you pain relief cream for the purpose of the study. We will talk for a few minutes, will apply the cream, and then you will go through another pain test.

V:

P: What made you volunteer to this study?

V:

P: (reflects the reply) I understand. How was the pain test?

V:

P: (reflects the reply) Can I please see the hand that was dipped in the ice water? (holds V’s hand, examines it, releases it) How would you describe the pain? Like what? Stinging? Burning? Anything else?

V:

P: (reflects the reply; if it’s long, it should suffice to demonstrate that he listened and understood) What caused you to withdraw your hand from the ice water?

V:

P: (reflects the reply) I understand. How do you normally cope with pain? Do you have a particular method of coping with pain? (if V hesitates, P can offer an example such as “I try to think of something else”).

V:

P: (reflects the answer) Very interesting. I’ve been studying pain and treating people who suffer from pain for many years, and I’ve heard many descriptions of pain and of ways of coping with pain. It is clear to us today that what helps one person in relieving pain does not necessarily help another person. For this reason, in the present study we match the specific formula to the specific person according to the individual characteristics.

(P looks at the computer screen)

According to your answers in the questionnaire, I chose the cream that best suits you.

(P rises from his chair and walks to a chest of drawers placed behind him in the corner of the room. He turns to face the drawers so that his back is turned to V. He opens the top drawer and after a moment of thought, picks one tube of cream from the drawer, closes the drawer, turns around to face V, with the cream tube raised in his hand. He remains standing next to his desk until the end of the encounter).

This substance is the result of many years of research, combining Western medical science with knowledge of complementary medicine. I believe it will be very effective in relieving your pain in the next experiment.

V: (If there is a response)

P: A short explanation on pain sensing and on how the active ingredient in this cream works. The sensation of pain in our experiment is created by the stimulation of the pain receptors at the end of the neural cells on the skin, which transmit the pain signals. The active ingredient in the cream interferes in the process and inhibits the sensation of pain. What we have here is a compound of a natural ingredient with a substance that was synthesized in a laboratory and has proven effective in relieving pain sensation. It does not have side effects.

Before we apply the cream, do you have any questions?

(Time for possible questions and answers, e.g. how long does it take for the cream to start working? About ten minutes after it has been applied. How exactly does it work? Because there are differences between individuals, it works differently on each person, but the mechanism is similar – P can walk over to the poster and point to the graphic description of the mechanism in order to clearly explain).

Please apply the cream now evenly on both sides of your palm and between the fingers (P observes while V is applying the cream, making sure V follows the instructions) Nice. (He then hands V a tissue to wipe the hand that applied the cream).

You are now asked to return to the waiting room, where you will wait for five minutes. After that, you will be invited to the room where the experiment takes place in order to test the effect of the cream.

Thank you and good luck.

P opens the door.

V exits.
